# Predicting Global Cognitive Decline in the General Population Using the Disease State Index

**DOI:** 10.3389/fnagi.2019.00379

**Published:** 2020-01-23

**Authors:** Lotte G. M. Cremers, Wyke Huizinga, Wiro J. Niessen, Gabriel P. Krestin, Dirk H. J. Poot, M. Arfan Ikram, Jyrki Lötjönen, Stefan Klein, Meike W. Vernooij

**Affiliations:** ^1^Department of Radiology and Nuclear Medicine, Erasmus MC University Medical Center Rotterdam, Rotterdam, Netherlands; ^2^Department of Epidemiology, Erasmus MC University Medical Center Rotterdam, Rotterdam, Netherlands; ^3^Department of Medical Informatics, Erasmus MC University Medical Center, Rotterdam, Netherlands; ^4^Department of Imaging Science and Technology, Faculty of Applied Sciences, Delft University of Technology, Delft, Netherlands; ^5^Department of Neurology, Erasmus MC University Medical Center Rotterdam, Rotterdam, Netherlands; ^6^VTT Technical Research Centre of Finland, Tampere, Finland; ^7^Combinostics, Tampere, Finland

**Keywords:** population based, cognitive decline, epidemiology, Disease State Index, prediction

## Abstract

**Background:**

Identifying persons at risk for cognitive decline may aid in early detection of persons at risk of dementia and to select those that would benefit most from therapeutic or preventive measures for dementia.

**Objective:**

In this study we aimed to validate whether cognitive decline in the general population can be predicted with multivariate data using a previously proposed supervised classification method: Disease State Index (DSI).

**Methods:**

We included 2,542 participants, non-demented and without mild cognitive impairment at baseline, from the population-based Rotterdam Study (mean age 60.9 ± 9.1 years). Participants with significant global cognitive decline were defined as the 5% of participants with the largest cognitive decline per year. We trained DSI to predict occurrence of significant global cognitive decline using a large variety of baseline features, including magnetic resonance imaging (MRI) features, cardiovascular risk factors, APOE-ε4 allele carriership, gait features, education, and baseline cognitive function as predictors. The prediction performance was assessed as area under the receiver operating characteristic curve (AUC), using 500 repetitions of 2-fold cross-validation experiments, in which (a randomly selected) half of the data was used for training and the other half for testing.

**Results:**

A mean AUC (95% confidence interval) for DSI prediction was 0.78 (0.77–0.79) using only age as input feature. When using all available features, a mean AUC of 0.77 (0.75–0.78) was obtained. Without age, and with age-corrected features and feature selection on MRI features, a mean AUC of 0.70 (0.63–0.76) was obtained, showing the potential of other features besides age.

**Conclusion:**

The best performance in the prediction of global cognitive decline in the general population by DSI was obtained using only age as input feature. Other features showed potential, but did not improve prediction. Future studies should evaluate whether the performance could be improved by new features, e.g., longitudinal features, and other prediction methods.

## Introduction

It is well established that neuropathological brain changes related to dementia accumulate over decades, and that the disease has a long preclinical phase. This may facilitate early disease detection and prediction (Jack et al., 2013). A large amount of literature on potential features and risk factors for dementia exists. However, clinicians often struggle to integrate all the data obtained from a single patient for diagnostic or prognostic purposes. Therefore, there is a need for information technologies and computer-based methods that support clinical decision making (Kloppel et al., 2008). Disease State Index (DSI) is a supervised machine learning method intended to aid clinical decision making (Mattila et al., 2011). This method compares a variety of patient variables with those variables from previously diagnosed cases, and computes an index that measures the similarity of the patient to the diagnostic group studied. The DSI method has previously been tested in specific patient populations and has shown to perform reasonably well in the early prediction of progression from mild cognitive impairment (MCI) to Alzheimer’s disease and has been successful in the classification of different dementia subtypes (Mattila et al., 2011, 2012; Munoz-Ruiz et al., 2014; Hall et al., 2015). In a recent study DSI has been validated in a population-based setting to predict late-life dementia (Pekkala et al., 2017). Identification of persons at risk for global cognitive decline may aid in early detection of persons at risk of dementia and may help to develop therapeutic or preventive measures to postpone or even prevent further cognitive decline and dementia (Blumenthal et al., 2017). This is especially important since previous research has shown that preventive interventions for dementia were more effective in persons at risk than in unselected populations (Moll van Charante et al., 2016). We therefore used DSI to predict global cognitive decline in the general population to select the persons at risk. The main aim of this study was to investigate whether multivariate data can predict global cognitive decline in the general population. If a high-risk group can be selected from the general population, a population screening program for this group might facilitate early detection of dementia. We evaluated the prediction performance using several sets of clinical features and brain magnetic resonance imaging (MRI) features, to assess whether the prediction is dependent on the combination of the input features. As brain MRI features we used all possible measures we could acquire: volumetric measures of gray matter, white matter, cerebrospinal fluid and white matter lesions, and a large variety of brain regions, both cortical as subcortical, diffusion measures, both globally as locally of a variety of tracts, cerebral blood flow measures, and the presence of microbleeds and infarcts. We used all these measures as we hypothesized that they could improve the prediction performance of cognitive decline. DSI was chosen as a classification method because this method is able to handle datasets with missing data, which is often the case in population study datasets. Also, this method has been successfully applied in previous studies and performed comparable to other state-of-the-art classifiers (Mattila et al., 2012; Pekkala et al., 2017).

## Materials and Methods

### Study Population

We included participants from three independent cohorts within the Rotterdam Study (RS), a prospective population-based cohort study in a suburb of Rotterdam, that investigates the determinants and occurrence of diseases in the middle-aged and elderly population (Ikram et al., 2017). Brain MRI-scanning was implemented in the study protocol since 2005 (Ikram et al., 2015). The Rotterdam Study has been approved by the medical ethics committee according to the Population Study Act Rotterdam Study, executed by the Ministry of Health, Welfare and Sports of the Netherlands. Written informed consent was obtained from all participants (Ikram et al., 2017). We used data from RS cohorts I, II, and III, of which each consists of multiple subcohorts. In this study a subcohort of RS cohort I, II, and III was used, to which we refer as sI, sII, and sIII, respectively. Baseline features of sI were collected during 2009–2011 and sII were collected during 2004–2006. The participants of the both cohorts were 55 years or older. For RS cohort III participants were 45 years or older at time of inclusion. Baseline features of sIII were collected during 2006–2008. Participants with prevalent dementia, MCI and MRI defined cortical infarcts at baseline were excluded for all analyses. In total, 4328 participants with baseline information on cognition, MRI and other features were included. Baseline MRI was acquired on average 0.3 ± 0.45 years after collecting the non-imaging features. Furthermore, diffusion-MRI was acquired. However, for a subset of 680 participants in RS cohort II diffusion-MRI data was obtained on average 3.5 ± 0.2 years later than the other baseline MRI features. Longitudinal data on global decline was available for 2,542 out of 4,328 participants. The follow-up cognitive assessment was on average 5.7 ± 0.6 years after the baseline visit.

### Disease State Index

Prediction was performed with DSI (Mattila et al., 2011). This classifier derives an index indicating the disease state of the participant under investigation based on the available features of that participant. DSI has two major advantages: (1) it can cope with missing data and (2) it gives an interpretable result because DSI also provides a decision tree that can be quite well explained.

Disease State Index classifier is composed of the components: fitness and relevance (Mattila et al., 2011). Let *N* be the total number of negatives, *P* the total number of positives, FN(*x*) the number of false negatives, and FP(*x*) the number of false positives, when *x* is used as classification cut-off. Then the fitness function is estimated for each feature *i* as:

$$f_{i}\,\left(x\right)=\frac{{\text{FNR}}_{i}\,\left(x\right)}{{\text{FNR}}_{i}\,\left(x\right)+{\text{FPR}}_{i}\,\left(x\right)}=\frac{{\text{FN}}_{i}\,\left(x\right)}{{\text{FN}}_{i}\,\left(x\right)+\frac{P}{N}\,{\text{FP}}_{i}\,\left(x\right)}$$

where FNR(*x*)=FN(*x*)/*P* is the false negative rate and FPR(*x*)=FP(*x*)/*N* is the false positive rate in the training data when the feature value *x* is used as the classification cut-off. The fitness automatically accounts for the imbalance in class size making implicitly both classes equal in size, as the fraction *P*/*N* in the denominator scales the negative class [related to FP(*x*)] to correspond the size of the positive class. The fitness function is a classifier where the values <0.5 imply negative class and >0.5 positive class. The relevance of each feature is estimated by:

R=max⁢{sensitivity+specificity-1, 0},

which measures how good the feature is in differentiating the two classes. The lower the overlap between the distributions of positives and negatives, the higher *R*. Finally, DSI is computed from the equation:

$$\text{DSI}=\frac{\sum_{i}R_{i}\,f_{i}}{\sum_{i}R_{i}}$$

**FIGURE 1:**
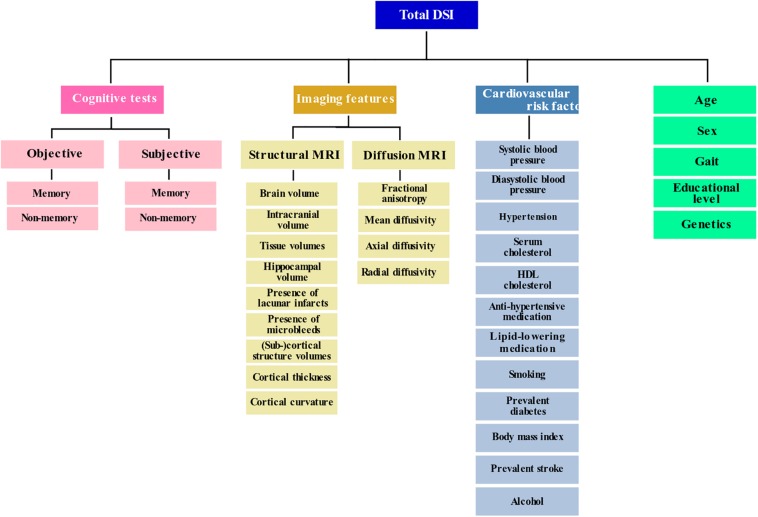
Feature categories shown in a hierarchy as used by the Disease State Index. Please note that not all individual features are included in this graph.

Disease State Index is a value between zero and one; somebody is classified as positive if DSI >0.5 and as negative if DSI <0.5. DSI is an ensemble classifier, meaning that it combines multiple independent classifiers (fitness functions) defined for each feature separately. Because of that, DSI can tolerate missing data. Features can be grouped in a hierarchical manner. The final DSI is a combination of the levels in the hierarchy. The fitness, relevance and their combination as a composite DSI are repeated recursively by grouping the data until a single DSI value is obtained. Therefore, the final DSI, which is used for the classification, depends on the hierarchy structure, as a different structure leads to a different averaging of the feature combinations. The top-level part of the hierarchy defined for this study is shown in Figure 1.

### Baseline Features

Figure 1 shows the used categories of features in hierarchical manner. Please note that not all individual features are shown in this figure. The sections below describe all the used features (indicated in bold font) in detail.

### MRI Features

Multi-sequence MR imaging was performed on a 1.5 Tesla MRI scanner (GE Signa Excite). The imaging protocol and sequence details were described extensively elsewhere (Ikram et al., 2015). Morphological imaging was performed with T1- weighted, proton density- weighted and fluid-attenuated inversion recovery (FLAIR) sequences. These sequences were used for an automated tissue segmentation approach to segment scans into gray matter, white matter, cerebrospinal fluid (CSF) and background tissue (Vrooman et al., 2007). Intracranial volume (ICV) (excluding the cerebellum and surrounding CSF cerebellar) was estimated by summing total gray and white matter and CSF. Brain tissue segmentation was complemented with a white matter lesion segmentation based on the tissue segmentation and the FLAIR image with extraction of white matter lesion voxels by intensity thresholding (de Boer et al., 2009). We obtained (sub)cortical structure volumes, cortical thickness, and curvature of the cortex and hippocampal volume using the publicly available FreeSurfer 5.1 software (Dale et al., 1999; Fischl et al., 2004; Desikan et al., 2006). For cerebral blood flow measurements, we performed a 2D phase-contrast imaging as previously described (Vernooij et al., 2008b). In short, blood flow velocity (mm/sec) was calculated based on regions of interest (ROI) drawn on the phase-contrast images in the carotid arteries and basilar artery at a level just under the skull base. The value of mean signal intensity in each ROI reflected the flow velocity with the cross-sectional area of the vessel. Flow was calculated by multiplying the average velocity with the cross-sectional area of the vessel (Vernooij et al., 2008b). A 3D T2^∗^-weighted gradient-recalled echo was used to image cerebral microbleeds. Microbleeds were defined as focal areas of very low signal intensity, smaller than 10 mm in size and were rated by one of five trained raters who were blinded to other MRI sequences and to clinical data (Roob et al., 1999; Vernooij et al., 2008a). Lacunar infarcts were defined as focal parenchymal lesions >3 mm and <15 mm in size with the same signal characteristics as CSF on all sequences and with a hyperintense rim on the FLAIR image (supratentorially). Probabilistic tractography was used to segment 15 different white matter tracts in diffusion-weighted MR brain images, and we obtained mean fractional anisotropy (FA), mean diffusivity (MD), axial and radial diffusivity inside each white matter tract (de Groot et al., 2015).

### Cardiovascular Risk Factors

Cardiovascular risk factors were based on information derived from home interviews and physical examinations during the center visit. Blood pressure was measured twice at the right brachial artery in sitting position using a random-zero sphygmomanometer. We used the mean of two measurements in the analyses. Information on the use of antihypertensive medication was obtained by using questionnaires and by checking the medication cabinets of the participants. Hypertension was defined as a systolic blood pressure >140 mmHg or a diastolic blood pressure >90 mmHg or the use of anti-hypertensive medication at baseline. Serum total cholesterol and high-density lipoprotein (hdL) cholesterol were measured in fasting serum, taking lipid-lowering medication into account. Smoking was assessed by interview and coded as never, former and current. Body-mass index (BMI) is defined as weight kilograms divided by height in meters squared. Diabetes mellitus status was defined as a fasting serum glucose level (>7.0 mmol/l) or, if unavailable, non-fasting serum glucose level (>11.1 mmol/l) or the use of anti-diabetic medication (Hofman et al., 2015). Alcohol consumption was acquired in a questionnaire. Prevalent stroke was ascertained as previously described (Akoudad et al., 2016). Educational level was assessed during a home interview and was categorized into seven categories, ranging from primary education only to university level (Hofman et al., 2015).

### APOE-ε4 Allele Carriership

APOE-ε4 allele carriership was assessed on coded genomic DNA samples. APOE- genotype was in Hardy- Weinberg equilibrium. APOE-E4 allele carriership was coded positive in case of one or two APOE-E4 alleles (Wenham et al., 1991).

### Gait Features

Gait was assessed by three walking tasks over a walkway: “normal walk,” “turn,” and “tandem walk” (heel to toe) (Lahousse et al., 2015). Using a principal component analysis we obtained the following gait factors which we used: rhythm, Variability, Phases, Pace, Base of Support, tandem, and turning (Verlinden et al., 2013).

### Baseline Cognitive Function

We included the following objective memory and non-memory cognitive tests: 15-word Learning test immediate and delayed recall (Bleecker et al., 1988), Stroop tests (reading, color-naming and interference) (Golden, 1976; Goethals et al., 2004), the Letter-digit Substitution task (Lezak, 1984), word fluency test (Welsh et al., 1994) and the Purdue Pegboard test (Desrosiers et al., 1995). Subjective cognitive complaints were evaluated by interview. This interview included three questions on memory (difficulty remembering, forgetting what one had planned to do, and difficulty finding words), and three questions on everyday functioning (difficulty managing finances, problems using a telephone, and difficulty getting dressed) (Hoogendam et al., 2014).

### Outcome: Definition of Cognitive Decline

A principal component analysis incorporating different cognitive tests was used to calculate a general cognitive factor (g-factor). For cognitive tests with multiple subtasks we chose only one subtask in order to prevent highly correlated tasks distorting the factor loadings. The following cognitive tests were included: color-word interference subtask of the Stroop test (which taps into information processing speed and executive functioning), LDST (testing executive function), verbal fluency test (tapping into executive functioning), delayed recall score of the 15-WLT (testing memory), and Purdue pegboard test (testing fine motor speed). The g-factor was identified as the first unrotated component of the principal component analysis and explained 49.2% of all variance in the cognitive tests. This is a typical amount of variance accounted for by the g-factor (Deary, 2012; Hoogendam et al., 2014). Cognitive decline was defined by the g-factor from the follow-up visit minus the g-factor from the baseline visit resulting in a delta g-factor. Since the follow-up time was not the same for each participant, the delta g-factor was divided by the follow-up time to obtain global cognitive decline per year. Significant global cognitive decline (yes/no) was defined as belonging to the 5% of participants with the highest cognitive decline (delta g-factor) per year. In the used dataset, consisting of 2,542 participants, this resulted in 127 participants with a positive class label.

### Evaluation Experiments

#### Prediction Performance Evaluation

The performance of DSI in predicting occurrence of global cognitive decline was evaluated using cross-validation. The area under the receiver-operator curve (AUC) was determined using 500 repetitions of 2-fold cross-validation (CV) experiments. This means that with each repetition 50% of the study dataset was used for training and the other 50% was used for testing, and vice versa, keeping the class ratio in the training and test set equal. We report the mean AUC, and the uncertainty of the mean expressed by its 95% confidence interval, derived from the 1,000 resulting AUC values. The confidence interval was determined with the corrected resampled *t*-test for CV estimators of the generalization error (Nadeau and Bengio, 2003). AUCs were considered significantly different if the 95% confidence interval of their difference did not contain zero.

Please note that the sample size was the same for baseline and follow-up, since we constructed a delta g-factor based on two time points. Only the people with cognitive assessment at both baseline and follow up were included in the analysis.

Since global cognitive decline per year is age dependent, we expect that age is an important feature for the prediction. We therefore include age as feature in the model. However, since other features might depend on age, correcting these features might improve the prediction performance (Falahati et al., 2016). We therefore also assessed the prediction performance using age-corrected features. We corrected the non-binary features for age using a linear regression model (Koikkalainen et al., 2012). We evaluated four different models:

(1) age was included and no age-correction was performed on the non-binary features
(2) age was excluded and no age-correction was performed on the non-binary features
(3) age was included and non-binary features, except age, were corrected for age
(4) age was excluded and non-binary features, except age, were corrected for age.

To assess whether the performance of DSI was dependent on the combination of input features, we evaluated various feature combinations. In each cross-validation experiment the feature set was expanded with a feature or category of features. We analyzed four of such cumulative feature sets, differing in the order in which the feature set was expanded. Additionally, we analyzed MRI features separately and a set including all features but age.

#### Relevance Analysis

To gain insight into the relevance weight that DSI assigns to each feature, we calculated the feature relevance distribution over the 500 repetitions of 2-fold CV, for the top-level feature categories of the hierarchy: age, sex, cognitive tests, cardiovascular risk factors, gait, education, genetics, and MRI features.

#### Feature Selection on MRI Features

In this study, hundreds of MRI features were extracted from images. It is likely that many of those features are not very efficient in detecting cognitive decline. Typically feature selection is applied to exclude poor features which may induce noise to the classifier. In DSI, weighting with relevance suppresses the effect of such features. If the number of features is high, their cumulative effect may, however, be remarkable. Previous results have shown that when including many features with a low relevance, the performance of DSI may decrease (Pekkala et al., 2017). We therefore included an experiment evaluating the effect of feature selection on MRI features using their relevance. Due to averaging, feature noise reduces in higher levels of the feature hierarchy. The relevance of top- level feature categories may therefore be higher than lower-level, individual features. Therefore, due to the selection on the individual features, the top-level features may drop out, despite their high relevance. To prevent entire top-level feature categories to drop out of the model, we chose to only apply feature selection on the MRI features, which made up 80% of all input features, before selection. The relevance of the MRI features was determined on the entire dataset, before training. MRI features were selected by thresholding the relevance. Subsequently, an AUC distribution was determined in 10 repetitions of 2-fold CV. The following relevance thresholds were chosen: *t*∈{0.0, 0.01,..., 0.09, 0.1}. For each threshold we assessed three feature sets in which the relevance-based feature selection on the MRI features was applied: (1) all features, (2) all features but age, and (3) MRI features only.

### Sub-Group Analyses

As subjects close to the decision boundary (DSI ∼0.5) are more likely to be misclassified, we evaluated classification performance when only accepting/providing the classification for test subjects with low (<0.2) or high (>0.8) DSI. In this way, the subjects with 0.2 < DSI < 0.8 are disregarded, which, in a clinical case, would mean that there is no diagnosis possible for these cases. We computed the AUC of this sub-group for DSI using all available features, both with age-correction and without age-correction. Furthermore, we performed a sensitivity analysis in which the diffusion-MRI of 680 participants in RS cohort II were ignored, because this data was obtained on average 3.47 ± 0.15 years later than the other baseline MRI features.

## Results

Table 1 presents the characteristics of the study population. The mean age of the participants was 60.9 ± 9.1 years and 55.6% were females. The absolute decline, given in an average difference in g-factor per year (standard deviation), was −0.25 (0.08) for the positive group (*N* = 127) and −0.02 (0.07) for the negative group (*N* = 2415). The threshold at 5% with the steepest decline was set at −0.18.

**TABLE 1:** Baseline features of the study population and their relevances.

Feature	*R*nac	*R*ac	Positive (*N* = 127)	Control (*N* = 2415)
Age, years	0.38	–	71.2 (10.1)	60.3 (8.7)
Sex, female	0.01	–	73 (54.5%)	1340 (55.6%)
Objective cognitive test results	0.28	0.16	–	–
Word Learning Test immediate recall	0.09	0.02	7.7 (2.2)	8.1 (2.0)
Word Learning Test delayed recall	0.05	0.04	7.9 (2.9)	8.2 (2.8)
Reading subtask of Stroop test, s	0.20	0.03	17.2 (2.7)	16.3 (2.9)
Color naming subtask of Stroop test, s	0.18	0.06	23.6 (3.6)	22.3 (4.0)
Interference subtask of Stroop test, s	0.32	0.10	53.8 (20.3)	44.0 (13.0)
Letter-Digit Substitution Task, number of correct digits	0.15	0.00	29.7 (6.7)	32.2 (6.2)
Word Fluency Test, number of animals	0.04	0.08	23.2 (5.7)	23.8 (5.7)
Purdue Pegboard test, number of pins placed	0.15	0.07	10.3 (2.1)	10.9 (1.7)
Mini-mental-state examination	0.14	0.11	27.8 (1.7)	28.4 (1.5)
Education^1^	0.07	0.07	3 (1-3)	3 (2-5)
Cardiovascular risk factors	0.34	0.27	–	–
Alcohol^1^, glasses per week	0.06	0.04	3.5 (0.3-5.5)	5.5 (1.0-5.5)
Systolic blood pressure, mmHg	0.24	0.04	146.2 (20.3)	135.9 (19.6)
Diastolic blood pressure, mmHg	0.00	0.02	82.8 (9.4)	82.4 (10.6)
Blood pressure lowering medication	0.26	–	51 (38.3%)	284 (11.9%)
Body Mass Index, kg/m^2^	0.07	0.07	28.2 (4.4)	27.4 (4.1)
Serum cholesterol, mmol/L	0.11	0.12	5.4 (0.9)	5.6 (1.1)
HDL-cholesterol, mmol/L	0.04	0.09	1.4 (0.4)	1.5 (0.4)
Lipid lowering medication	0.13	–	46 (34.6%)	510 (21.3%)
Smoking	0.08	0.08	–	–
Never	–	–	49 (36.6%)	746 (31.2%)
Former	–	–	54 (40.3%)	1154 (48.2%)
Current	–	–	31 (23.1%)	492 (20.6%)
Diabetes mellitus, presence	0.09	–	24 (18.2%)	220 (9.2%)
APOE-E4 allele carriership	0.02	–	39 (30.2%)	639 (28.3%)
MRI features	0.41	0.25	–	–
Intra-cranial volume, mL	0.03	0.00	1137 (119)	1144 (113)
Brain tissue volume	0.38	0.08	–	–
White matter volume, mL	0.13	0.01	390 (60)	419 (57)
Gray matter volume, mL	0.10	0.01	522 (54)	537 (52)
CSF volume, mL	0.29	0.07	223 (53)	186 (46)
Brain region volume	0.35	0.12	–	–
Hippocampus volume, mL	0.23	0.09	6.4 (0.8)	6.8 (0.7)
White matter lesion volume^1^, mL	0.31	0.08	5 (2.5-9.4)	2.4 (1.4-4.3)
Cerebral microbleeds, presence	0.09	–	33 (24.6%)	370 (15.6%)
Lacunar infarcts, presence	0.04	–	10 (7.5%)	72 (3.0%)
Global FA	0.17	0.07	0.3 (0.02)	0.3 (0.01)
Global MD, 10^–3^ mm^2^/s	0.33	0.07	0.8 (0.03)	0.7 (0.03)
Global cortical thickness, mm	0.08	0.01	2.4 (0.2)	2.5 (0.1)
Gait	0.19	0.17	–	–

*The relevances were computed on the entire dataset. Continuous variables are presented as mean (standard deviation) and categorical variables as *n* (percentages). N, number of participants; HDL, high-density lipoprotein; s, seconds; FA, fractional anisotropy; MD, mean diffusivity 10-3 mm^2^/s. CSF, cerebrospinal fluid. Symbols: *R*nac; relevance when feature was not corrected for age, *R*ac; relevance when feature was age-corrected. 1Education, alcohol and white matter lesion volume are presented as median (inter-quartile range).*

### Prediction Performance

**FIGURE 2:**
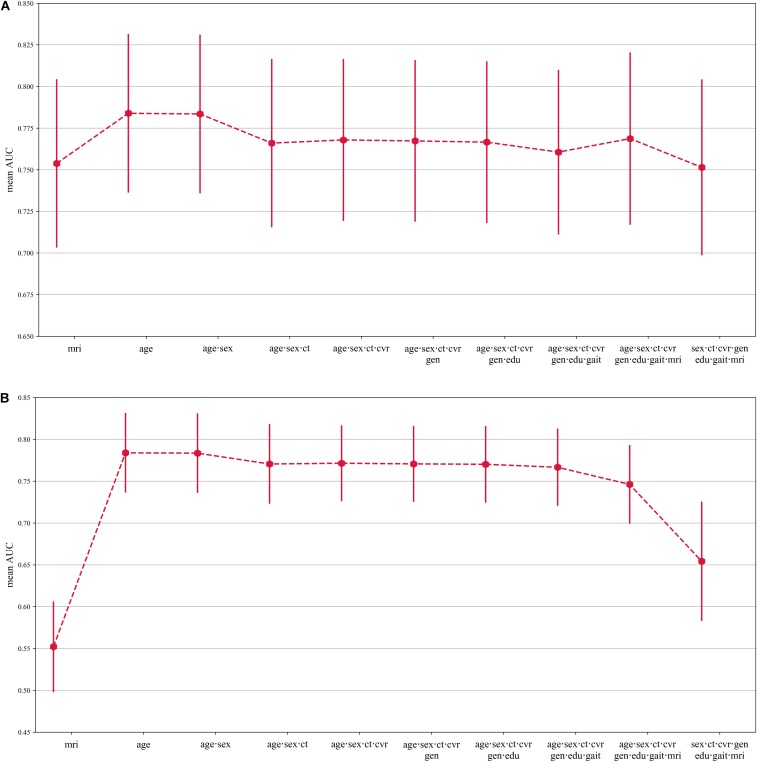
**(A)** Graphs showing the AUC and 95% confidence interval for classification of cognitive decline for different combinations of features in the DSI model. Other feature combinations are not shown, since they were not significantly different from the feature combinations shown in this figure. The features have *not* been corrected for age. AUC performance for the different combinations of features are shown on the *y*-axis. Abbreviations are used for the various features: cognitive tests (ct), cardiovascular risk factors (cvr), MRI features (mri), genetics (APOE-E4 carrier-ship) (gen), and educational level (edu). Note that the *y*-axis scale ranges from 0.65–085. **(B)** Graphs showing the AUC and 95% confidence interval for classification of cognitive decline for different combinations of features in the DSI model. Other feature combinations are not shown, since they were not significantly different from the feature combinations shown in this figure. The binary features have been corrected for age. AUC performance for the different combinations of features are shown on the *y*-axis. Abbreviations are used for the various features: cognitive tests (ct), cardiovascular risk factors (cvr), MRI features (mri), genetics (APOE-E4 carrier-ship) (gen), and educational level (edu). Note that the *y*-axis scale ranges from 0.45–085.

Figure 2A shows the mean AUC (95% confidence interval) for several combinations of features in predicting global cognitive decline, without correcting the non-binary features for age. Other feature combinations are not shown, since they were not significantly different from the feature combinations shown in Figure 2A. When using only MRI features, the AUC was 0.75 (0.70–0.80). When using only age as baseline feature, the AUC was 0.78 (0.74–0.83). Using additional features on top of age resulted in an equal or slightly lower AUC (differences not statistically significant). When using all available features with DSI, the AUC was 0.77 (0.72–0.82). The mean AUC of DSI without age as baseline predictor was 0.75 (0.70–0.80).

Figure 2B shows the mean AUC (95% confidence interval) for the same combinations of features as in Figure 2A, but here the non-binary features were corrected for age. The AUC for MRI features only was significantly lower with age-correction compared to without age correction, with an AUC of 0.55 (0.50–0.61). For the other feature sets, the AUC of the models where age correction was applied was not statistically significantly different, compared to not using age correction. When the effect of age was totally removed from the model, i.e., model IV, the AUC was 0.65 (0.58–0.73).

### Relevance Analysis

**FIGURE 3:**
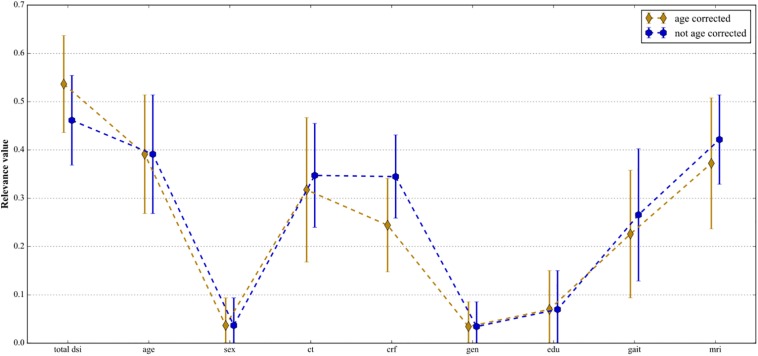
Mean relevance weight *R* and 95% confidence interval for the top-level features categories: total DSI, age, sex, cognitive tests, cardiovascular risk factors, genetics, education, gait, and MRI features. The blue line shows the case where the non-binary features were *not* corrected for age and the golden line shows the case where the non-binary features were age-corrected.

Figure 3 shows the relevance weight per feature category when the non-categorical features were corrected for age prior to computing DSI and without age-correction. Without age-correction, the features with the best discriminating abilities according to their relevance weights were MRI features [0.42 (0.33–0.51)], age [0.39 (0.27–0.51)], cognitive tests [0.35 (0.24–0.45)] and cardiovascular risk factors [0.34 (0.26–0.43)]. When correcting the non- binary features, except age, for age, the most discriminating features were age 0.39 (0.27–0.51)], MRI features [0.37 (0.24–0.51)], and cognitive tests [0.32 (0.17–0.47)].

### Feature Selection on MRI Features

**FIGURE 4:**
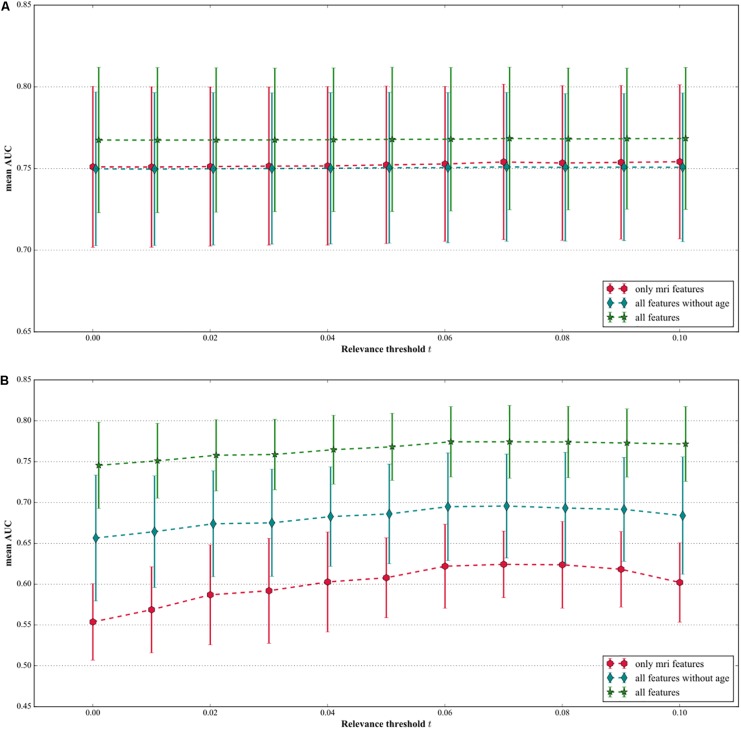
**(A)** Mean AUC and 95% confidence interval for several combinations of features where the MRI features were selected based on their relevance without correcting the non-binary features for age. Feature selection does not improve the AUC and there is no difference between all features without age and MRI features only. **(B)** Mean AUC and 95% confidence interval for several combinations of features where the MRI features were selected based on their relevance where the non-binary features were corrected for age.

Feature selection for MRI features had no effect on the AUC in any of the three feature sets, when the non-binary features were not corrected for age (Figure 4A). The AUC did increase after MRI feature selection when the non-binary features, except age, had been corrected for age, with the optimal *t* being 0.07 (see Figure 4B). For *t* = 0.07, the AUC increased from 0.55 (0.50–0.61) to 0.62 (0.58–0.67) when only MRI features were included in the model. When using all features, the AUC increased from 0.75 (0.70–0.79) to 0.77 (0.73–0.82), and when using all features but age, the AUC increased from 0.65 (0.58–0.73) to 0.70 (0.63–0.76).

### Sub-Group Analyses

When only taking into account the extreme cases, i.e., cases for which 0.2 < DSI < 0.8 (∼40% of the total dataset, i.e.,∼1000 subjects), the mean AUC increased to 0.82 (0.76–0.88) using age as input feature only. Again in this group, additional features did not significantly improve the performance of DSI (results not shown). Ignoring the diffusion-MRI features of 680 participants of whom this data was acquired on average 3.47 ± 0.15 years later than the assessment of the other baseline MRI features did not change AUC significantly compared to the performance in the total population (results not shown).

## Discussion

The objective of this study was to assess whether global cognitive decline can be predicted using multivariate data with the previously proposed DSI. We found the best prediction performance, evaluated with AUC, using only age as input feature. Adding more features to DSI did not improve its performance in predicting global cognitive decline as defined in this study. Overall performance of DSI in the prediction of global cognitive decline (mean AUC 0.78) was comparable to previously reported performances of DSI for prediction of dementia in the population-based CAIDE study, consisting of 2,000 participants who were randomly selected from four separate, population-based samples, originally studied in midlife (1972, 1977, 1982, or 1987) (Rusanen et al., 2014) and to other population-based prediction models of dementia (Kivipelto et al., 2006). In this study we included a large number of heterogeneous features. Age was the most important feature for predicting global cognitive decline using DSI, yielding the highest AUC. This was further supported by the observation that the performance of DSI reduced when using all features except age. Our finding that age is the single strongest predictor for cognitive decline is in line with published prediction models for dementia, that invariably assign the highest weight to age (Stephan et al., 2015; Park et al., 2019). Also, a recent paper by Licher et al. (2018) which validated four dementia prediction models in the general population also concluded that the discriminatory ability of different models was as good as using age alone. We found that the relevance R, which indicates how well a feature can discriminate between persons who will develop cognitive decline and those who will not, was highest for MRI features (0.42) followed by age (0.39). DSI, however, performed worse when using only MRI features, compared to using only age. We speculate that the high relevance of the MRI features may be explained by age-specific effects that are captured in these MRI features, which is supported by our finding that MRI feature relevance (0.37) and DSI performance dropped when adjusting MRI features for age. When the non-binary features were age-corrected and age was not included in the model, the mean AUC was 0.65, still significantly better than chance (0.5), indicating that relevant information for predicting global cognitive decline could be present in the other features. In this study, however, they did not improve the predicting performance when added to age. To our surprise we found that APOE-ε4 allele carrier-ship had a low relevance weight and did not improve the performance of DSI, even though it is the best known genetic risk factor for AD. This is in contrast to a previous study focusing on the progression from MCI to AD, which found APOE-genotype to have high predictive value (Lowe et al., 2016). It may be that our study population was too young to show an effect of APOE on prediction (mean age 60.9), since the risk progression effect of APOE-ε4 allele carriership has been described to peak between ages 70 and 75 years (Bonham et al., 2016). The relevance-based feature selection on the MRI features showed an increase in the AUC, but only when the non-binary features were corrected for age. A possible explanation is that without age correction, the AUC is strongly driven by the age-factor that is present in the MRI features.

In this case, less and different features were excluded compared to the age-corrected models, causing the selection to have no effect on the prediction performance. However, after removal of these age-specific effects by age correction, performance can be increased by removal of irrelevant features. When age was totally excluded from the model IV (age was excluded and age correction was applied to the non-binary features), an AUC of 0.70 was obtained, showing the potential of the other features. One limitation of this analysis is that the relevance computation and threshold selection was done on the entire dataset, i.e., the training data was included in these computations. Therefore, AUC increase due to application of the relevance threshold might be overestimated, but can be seen as an upper limit. The overall conclusions do not change.

To our knowledge, this is the first population-based study testing the supervised machine learning DSI tool for prediction of global cognitive decline. We did not choose dementia as outcome as the predictive power would be too low, since there were too few cases of dementia in the used dataset as we focused on a non-demented population. We also did not choose MCI as outcome. The diagnosis of MCI may be less reliable in a population-based setting, as it is a clinical diagnosis which is strongly dependent on a subject seeking clinical care for experienced subjective cognitive complaints (Edmonds et al., 2016). There have been previous attempts to diagnose MCI in the setting of The Rotterdam Study, but the MCI diagnosis definition used in this context is based on a number of baseline features that were used as predictor in our study. It would therefore not have been statistically sound to use MCI as an outcome, given our set of predictors. Furthermore, in a general population and for application in a first line setting, it may be most helpful to more broadly predict which individuals may experience a higher than expected rate of global cognitive decline in the future.

Strengths of our study include the population-based design, large sample size and availability of an extensive set of features. However, limitations of our dataset need to be considered. We constructed a g-factor as a measure of global cognition and participants without complete cognitive data were excluded. This might have caused some selection bias toward relatively healthy subjects. Also, mortality and drop-out was not taken into account. Persons who are lost to follow-up usually have a poorer health status and are therefore more likely to develop cognitive decline or die before onset of cognitive decline. The exclusion of these assumingly more severe cases might have lowered the performance of DSI. The result that age is the main predictor for cognitive decline indicates that the age distribution of the subjects with cognitive decline differs from the entire set of subjects. Hence age could be used to select people at risk of cognitive decline. However, when screening for significant cognitive decline, an age-dependent threshold on cognitive decline might be needed, e.g., using the 5% percentile of the cognitive decline as function of age, to detect young people at risk of developing dementia. The usage of such an age-dependent threshold will be part of future research. Finally, it should be noted that cognitive decline is not equivalent to neurodegeneration/dementia and may result from other causes as well, due to conditions affecting the participant’s cognition at the time of the cognitive assessment, normal human variability and normal aging. Nevertheless, being able to predict cognitive decline would be a step forward in selecting people for therapy or prevention.

## Conclusion and Future Work

Based on our results we can conclude that age is the most important predictor for cognitive decline in the general population using DSI. Other features showed potential, but did not improve prediction performance. A next step could be to use longitudinal features in DSI, as these might improve its prediction performance. To validate whether our findings are not due to limitations of DSI, also other methods need to be evaluated in this prediction challenge. Finally, to be able to detect younger people at risk of significant global cognitive decline in future studies, thresholds for cognitive decline should be carefully chosen depending on the population, for example be age-adjusted.

## Data Availability Statement

The datasets generated for this study are available on request to the corresponding author.

## Ethics Statement

The studies involving human participants were reviewed and approved by the Medical Ethics committee according to the Population Study Act Rotterdam Study executed by the Ministry of Health, Welfare and sport of the Netherlands. The patients/participants provided their written informed consent to participate in this study.

## Author Contributions

WN, MV, SK, GK, JL, DP, and MI designed the study, provided the funding, and supervision. LC and WH collected and analyzed the data, had full access to all the data in the study, and take responsibility for the integrity of the data and the accuracy of the data analysis. LC, WH, SK, DP, WN, JL, MV, and MI interpreted the results and drafted the manuscript. All authors critically revised the manuscript for important intellectual content and meet all four criteria for authorship according to the guidelines of the International Committee of Medical Journal Editors, as revised in 2013.

## Conflict of Interest

JL was employed by the company Combinostics, which develops advanced tools for data-driven diagnostics. WN was employed by the company Quantib B.V, which develops software to extract quantitative imaging biomarkers from medical imaging data. GK received research grants from GE Healthcare, Siemens AG and Bayer Health Care, Bracco, € 40.000 per year consultancy fee. The remaining authors declare that the research was conducted in the absence of any commercial or financial relationships that could be construed as a potential conflict of interest.
